# Anti-rotavirus antibody measurement in a rotavirus vaccine trial: Choice of vaccine antigen in immunoassays does matter

**DOI:** 10.1080/21645515.2023.2167437

**Published:** 2023-01-30

**Authors:** Xiaoqian Wang, Daniel E. Velasquez Portocarrero, Margaret M. Cortese, Umesh Parashar, Khalequ Zaman, Baoming Jiang

**Affiliations:** a Division of Viral Diseases, Centers for Diseases Control and Prevention (CDC), Atlanta, GA, USA; b Division of Infectious Diseases, International Centre for Diarrhoeal Disease Research, Bangladesh (icddr,b), Dhaka, Bangladesh

**Keywords:** Rotavirus, vaccine, immunogenicity, enzyme immunoassay

## Abstract

In a clinical trial of Bangladeshi infants who received three doses of RotaTeq (ages 6, 10, and 14 weeks), we did a head-to-head assessment of two vaccine virus strains to measure rotavirus IgA in sera. Serum samples collected at pre-dose 1 (age 6 weeks) and post-dose 3 (age 22 weeks) were tested for rotavirus IgA by EIA by using the matching vaccine strain (RotaTeq) and a different vaccine strain (Rotarix) as antigens. Overall, rotavirus IgA seropositivity and titers with each antigen were compared. At age 22 weeks (N = 531), the proportion of infants who tested rotavirus IgA seropositive was similar when measured using the RotaTeq (412/531 [78%]) or the Rotarix antigen (403/531 [76%]) in the EIA. However, the IgA geometric mean titer was higher when measured using the RotaTeq antigen as compared to that measured using the Rotarix antigen [218 (95%CI: 176–270) vs. 93 (77–111), *p* < .0001]. We have compared two globally licensed vaccines, the human monovalent, and the pentavalent reassortant, as antigens on a RotaTeq cohort, resulting in higher estimations of IgA antibodies in the same sample when measured using the RotaTeq antigen. Our findings support matching vaccine antigens in EIA for the most desired immunogenicity testing of the RotaTeq vaccine.

## Introduction

Globally, rotavirus infections were responsible for estimated 151 514 deaths in children younger than five years in 2019, along with millions of cases and hospitalizations.^1^ Rotavirus vaccines have been administered to millions of children worldwide.^2^ Rotarix (monovalent attenuated, G1P[8]; GlaxoSmithKline),^3^ RotaTeq (pentavalent human-bovine reassortants G1, G2, G3, G4, P[8]; Merck),^4^ Rotavac (Monovalent neonatal G9P[11]; Bharat) and RotaSiil (pentavalent human-bovine reassortants G1, G2, G3, G4, G9; Serum Institute of India), were recommended by World Health Organization for all children.^5^ Other rotavirus vaccines such as Rotavin-M1 (monovalent attenuated G1P[8]) and the Lanzhou lamb vaccine (monovalent lamb G10P[12]) have been licensed for domestic use in the private market in Vietnam and China, respectively.^6^ Additionally, several candidate parenteral rotavirus vaccines are currently in different stages of development.^7^

There is evidence that serum rotavirus IgA is associated with clinical protection at the individual and population levels.^8,9^ IgA antibodies do not cross the placenta. They therefore are not present in the blood of infants unless a natural infection has occurred or the infant has received a rotavirus vaccine. Since concurrent efficacy trials of various rotavirus vaccines in the same setting are challenging for financial and ethical reasons, comparisons of the performance are often based on immunogenicity evaluations. Accordingly, reliable rotavirus IgA EIA is crucial for measuring the immunogenicity of oral rotavirus vaccines. However, serologic analyses in clinical trials have used both the homotypic and heterotypic antigens indistinctively. There is no consensus regarding using rotavirus strain or antigen type in EIA across studies.^10,11^ To facilitate research in choosing appropriate antigens for each vaccine serological testing, we assessed the immunogenicity of RotaTeq in a clinical trial in Bangladesh using two vaccine strains (the matching RotaTeq strain and non-matching Rotarix strain) as antigens in a rotavirus IgA EIA.

## Methods

### Study design and procedures

As part of a randomized, controlled, open-label, parallel, phase 4 trial in Mirpur and Mohakhali in Dhaka, Bangladesh, one group of infants received three doses of the oral RotaTeq rotavirus vaccine at ages 6, 10, and 14 weeks (study registered with ClinicalTrials.gov, number NCT02847026).^12^ The study protocol number PR-15105 was approved by the Institutional Review Board and Centre Director of the International Center for Diarrhoeal Disease Research, Bangladesh (icddr,b). The protocol was shared with the U.S. Centers for Disease Control and Prevention (CDC) but deferred to the icddr,b’s Institutional Review Board. Written informed consent was obtained from the parents of all participants. Serum was collected at pre-specified time points, and samples used in this report were collected at age 6 weeks (before RotaTeq dose 1) and age 22 weeks (8 weeks after RotaTeq dose 3 at age 14 weeks). All serum samples were stored at −20°C at the study clinic and transported at the end of each day to the icddr,b laboratory to be stored at −20°C. Samples were shipped to the US CDC (Atlanta, USA) for testing. Rotavirus IgA was performed using an EIA.^12,13^ Briefly, the 96-well microplate wells coated with 100 µl/well of rabbit hyperimmune serum to the rhesus rotavirus strain RRV at a 1:10,000 dilution, then incubated overnight. Microplates were incubated with 200 µl/well of blocking buffer (5% blotto in PBS) for 1 hour. Microplates were incubated for 1 hour in duplicate with 100 µl/well clarified lysates of either RotaTeq G1 strain or Rotarix strain-infected MA104 cell cultures in working dilutions of 7.2 × 10^5^ FFU/mL and 7.2 × 10^5^ FFU/mL for RotaTeq and Rotarix antigens, respectively. 100 µl/well of serial dilutions of the test sera (1:20–1:10,240; 100 µl/well) were added, and plates were incubated for 2 hours, followed by adding 100 µl/well of biotin-conjugated goat anti-human IgA (KPL, USA) diluted 1:2,000 and incubated for 1 hour. Extravidin (100 µl/well) (Sigma, USA) diluted 1:3,000 was added to the wells and incubated for 1 hour, and then the reactions were developed with 100 µl/well 3,30,5,50-tetramethylbenzidine (Sigma, USA) and stopped with 100 µl/well 1 N HCl. Absorbance was subsequently read at 450 nm by an ELISA plate reader (Dynex). Rotavirus IgA titers in serum were calculated as the reciprocal of the highest dilution that gave a mean OD greater than the cutoff value (3 standard deviations above the mean OD of the negative control serum wells).

### Outcomes and statistical analysis

The outcome was the comparison of the rotavirus IgA seroresponses measured in serum collected at age 22 weeks (after completing the 3-dose RotaTeq vaccine series at age 14 weeks) when performed with RotaTeq antigen EIA vs. performed with Rotarix antigen EIA. Rotavirus IgA seroresponse was measured in three ways: rotavirus IgA seropositivity, rotavirus IgA seroconversion, and rotavirus IgA titers or geometric mean titers (GMT). Rotavirus IgA seropositivity was defined as a titer of 40 or greater. Rotavirus IgA seroconversion was defined as IgA seropositivity (titer ≥40) at age 22 weeks if seronegative (titer <40) at age 6 weeks or a four-fold or greater increase in rotavirus IgA titer in the subsequent sample if rotavirus IgA seropositive (titer ≥40) at age 6 weeks when using the same antigen at age 6 weeks and age 22 weeks. The GMT was defined as the exponential mean logarithmic transformation of the rotavirus IgA titers. The log-transformed antibody titers were compared between the RotaTeq and Rotarix antigen groups using one-way ANOVA with Dunnett’s multiple-comparison correction. Proportions were compared between groups using Fisher’s exact test. Spearman rank correlations were used to examine relationships between the log-transformed rotavirus IgA titers from the two different antigens. A two-sided α and significance level of 0.05 was used for all statistical tests. Data were analyzed in SPSS (version 21).

## Results

**Figure 1.**
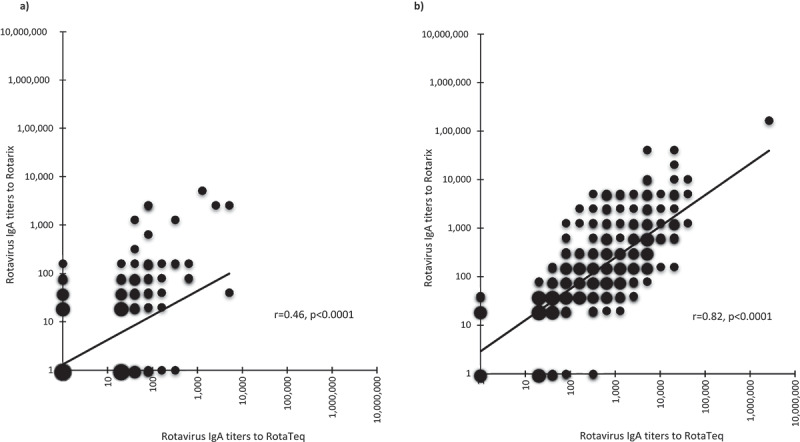
Association of serum Rotavirus IgA titers measured using RotaTeq or Rotarix antigen in EIA (n = 531 infants) at age 6 weeks (a) and at age 22 weeks (b).

Between September 1 and December 8, 2016, 571 infants in the trial were randomized to receive RotaTeq, and 531 completed the study and had testable serum samples. At age 6 weeks, the proportion of infants who tested rotavirus IgA positive was similar when measured via EIA using RotaTeq G1 antigen (67 [13%] of 531) or Rotarix antigen (69 [13%] of 531) (Table 1). Also, RotaTeq and Rotarix antigen assays gave similar rotavirus IgA GMT in the total [4 (95% CI: 3–4) vs. 3 (2–3)] and in the seropositive infants [n = 67, 90 (67–120) vs. n = 69; 100 (74–134)]. At age 22 weeks, the proportion of infants who tested IgA-positive was similar when measured using the RotaTeq antigen assay (412 [78%] of 531) or the Rotarix antigen assay (403 [76%] of 531), and the proportion of infants with seroconversion was also similar when measured with either antigen assay (Table 1). However, the rotavirus IgA GMT was higher when measured using the RotaTeq G1 antigen as compared to that measured using the Rotarix antigen for all infants combined [218 (176–270) vs. 93 (77–111), *p* < .0001]. Similarly, rotavirus IgA GMT was higher when assessed for only the IgA-seropositive infants [549 (456–662) vs. 210 (179–245), *p* < .0001], or when assessed for only the infants who had IgA seroconversion [574 (473–696) vs. 222 (188–262), *p* = .0004]. Additionally, at age 22 weeks, although the calculated rotavirus IgA titers were higher when the RotaTeq G1 strain was used as antigen, the Spearman’s correlation coefficient between the two assays showed a strong positive correlation with an r-value of 0.82 (*p* < .0001), demonstrating a very strong correlation (Figure 1). At the individual patient level, there was discordance in seropositivity results between the two antigens EIAs in 54 [10%] and 71 [13%] out of 531 infants at age 6 and 22 weeks, respectively.

Log-transformed rotavirus IgA titers in paired RotaTeq or Rotarix antigen EIA in infant sera were analyzed using Spearman correlation analysis. The color intensity and circle size at each point were proportional to the number of samples.

**Table 1.** Serum rotavirus IgA result pre (age 6 weeks) and post- (age 22 weeks) RotaTeq vaccine series measured using RotaTeq or Rotarix antigen in EIA (n = 531 infants).

Age (weeks)	Endpoint	Rotavirus IgA result using RotaTeq antigen in EIA	Rotavirus IgA result using Rotarix antigen in EIA	*p* value	Rotavirus IgA positivity with both RotaTeq antigen and Rotarix antigen in EIAs	Rotavirus IgA positivity with either RotaTeq antigen or Rotarix antigen in EIAs
6 ^(1)^	RV-IgA GMT total (95%CI)	4 (3–4)	3 (2–3)	.52		
	RV-IgA positivity: n/N; %	67/531; 13%	69/531; 13%	.93	41/531; 8%	95/531; 18%
	RV-IgA GMT of seropositives (95%CI)	90 (67–120); n = 67	100 (74–134); n = 69	.61		
22^(2)^	RV-IgA GMT total (95%CI)	218 (176–270)	93 (77–111)	<.0001		
	RV-IgA positivity: n/N; %	412/531; 78%	403/531; 76%	.56	372/531; 70%	443/531; 83%
	RV-IgA GMT of seropositives (95%CI)	549 (456–662); n = 412;	210 (179–245); n = 403	<.0001		
	RV-IgA seroconversion: n/N; %	394/531; 74%	370/531; 70%	.12	340/531; 64%*	442/531; 83%*
	RV-IgA GMT of seroconverters (95%CI)	574 (473–696); n = 394	222 (188–262); n = 370	.0004		

Data are n (%) or GMT (95% CIs), unless otherwise specified. GMT = geometrical mean titers. ^(1)^Before first dose. ^(2)^8 weeks post dose 3 of RotaTeq. RV = Rotavirus.

*Rotavirus IgA seroconversion was defined as IgA seropositivity (titer ≥40) at age 22 weeks if seronegative (titer <40) at age 6 weeks or a four-fold or greater increase in rotavirus IgA titer in the subsequent sample if rotavirus IgA seropositive (titer ≥40) at age 6 weeks, when using the same antigen at age 6 weeks and age 22 weeks.

## Discussion

We showed that the RotaTeq and the Rotarix antigen EIAs produced similar overall seropositivity and seroconversion results on a large panel of serum samples collected from infants after receiving a full series of RotaTeq. However, due to specific antigen-antibody interactions, the RotaTeq antigen EIA produced higher rotavirus IgA GMT than the Rotarix antigen. A previous study showed that in RotaTeq recipients, a higher rotavirus IgA positivity, and GMT were achieved using WC3-antigen (RotaTeq parent) assay compared to 89–12 antigen (Rotarix parent) assay.^14^ They also showed that in Rotarix recipients, a higher rotavirus IgA positivity, and GMT were achieved using 89–12 antigen assays compared to a WC3-antigen assay. These results indicate that using specific vaccine antigens (e.g., Rotarix vaccine-Rotarix antigen, RotaTeq vaccine-RotaTeq antigen) might be the desired option for rotavirus vaccine immunogenicity studies.

Although the overall levels of seropositivity among all subjects were similar using either antigen in the assay, we observed some discordance in the seropositivity at the individual level when measured using different vaccine strains. The 10% discrepancy at age 6 weeks might be due to natural infection from rotavirus genotypes that differ from the vaccine antigens used. Meanwhile, the 13% discrepancy observed at age 22 weeks might be due not only to natural infection but also to a higher specific antigen-antibody affinity provided by the RotaTeq antigen in this RotaTeq immunized cohort. Our study has limitations. We use rotavirus IgA as a surrogate of protection; however, rotavirus IgA seroresponse is not considered a direct proxy for efficacy. We tested rotavirus IgA titers with two vaccine antigens in the RotaTeq cohort, which could not represent the results of the Rotarix immunized arm that was part of this clinical trial.^12^

In conclusion, we evaluated the performance of two vaccine strains in EIA, which achieved comparable positivity but yielded different rotavirus IgA titers due to strain-specific host response. Rotavirus antibody quantification greatly depends on the methods and antigens used to assess immune response in vaccine trials. Hence, it is important for vaccine manufacturers and researchers to understand assay performance and use specific rotavirus strains as antigens to determine antibody concentrations in immunogenicity evaluation. Our results show that the antigen type used in the EIA should match the vaccine strain under study for the most desired evaluation of RotaTeq immunogenicity in children.
